# Supplementary material for: Short-term exposure to wildfire-related PM2.5 increases mortality risks and burdens in Brazil
Source: Nat Commun. 2022 Dec 10;13:7651. doi: 10.1038/s41467-022-35326-x (PMC9741581; doi:10.1038/s41467-022-35326-x)
Supplement: Supplementary file 1 — Supplementary Information [file 41467_2022_35326_MOESM1_ESM.docx]

**Supplemental Material**

**Short-term exposure to wildfire fine particulate matter and mortality risks and burdens in Brazil: A nationwide time-series study between 2000 and 2016**

| **Table of Contents** |  | **Page** |
| --- | --- | --- |
| **Supplementary Figures** |  |  |
| **Supplementary Figure 1**. The immediate-region-level (n = 510) daily wildfire-related PM_2.5_ in different months from 2000 to 2016. |  | **2** |
| **Supplementary Figure 2**. The linearity of the relationship between daily wildfire-related PM_2.5_ and all-cause, cardiovascular, and respiratory mortalities across lag 0–7 days. |  | **3** |
| **Supplementary Figure 3.** The sex differences within each age subgroup. |  | **4** |
| **Supplementary Figure 4**. Lag patterns with different maximum lag day. |  | **5** |
| **Supplementary Figure 5.** The framework of statistical analysis. |  | **6** |
|  |  |  |
| **Supplementary Tables** |  |  |
| **Supplementary Table 1.** Results of sensitivity analyses changing maximum lag days and the df of lag days for wildfire-related PM_2.5_, and the df of lag days for daily mean temperature. |  | **7** |


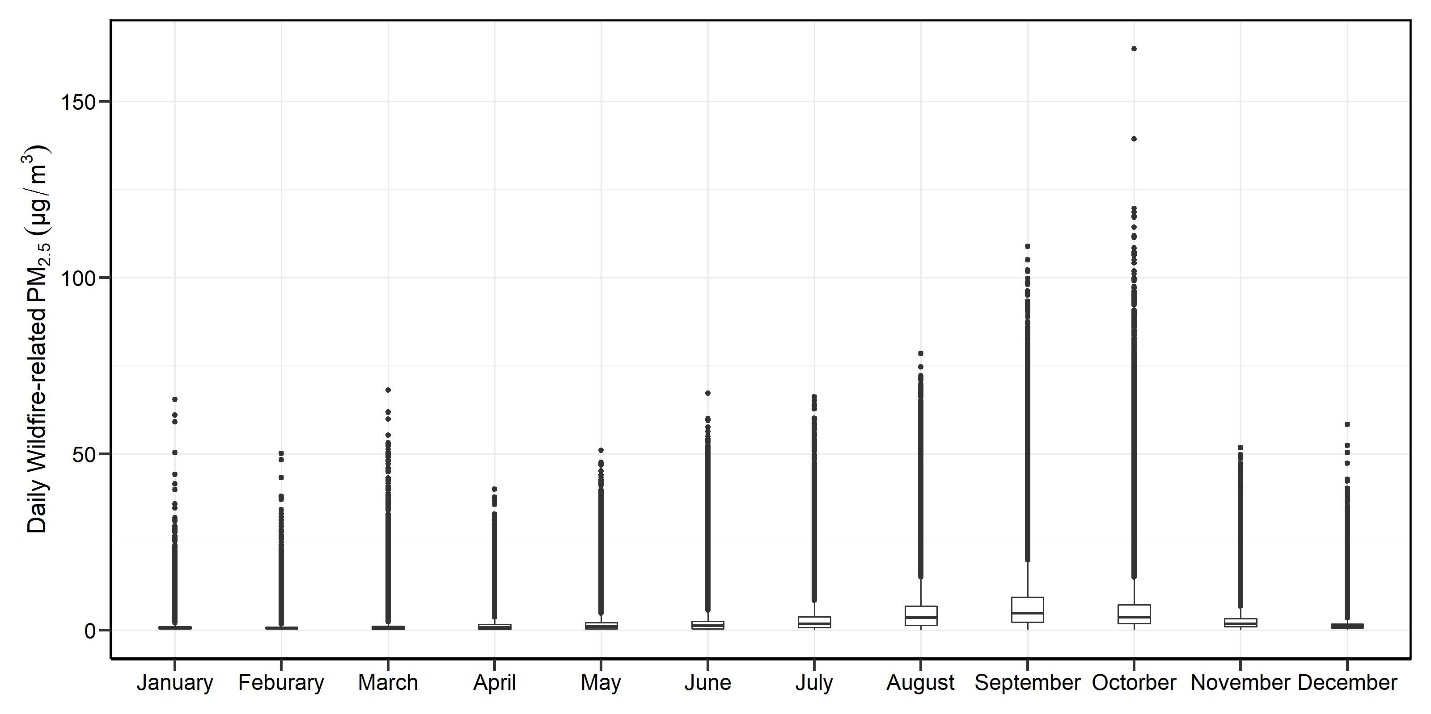


**Supplementary Figure 1**. The immediate-region-level (n = 510) daily wildfire-related PM_2.5_ in different months from 2000 to 2016. (Note 1: The elements in the box plots represent the median, the interquartile range (black rectangles) and the outside values in the datasets (black dots). Note 2: PM_2.5_, fine particulate matter with diameter ≤ 2.5 μm).


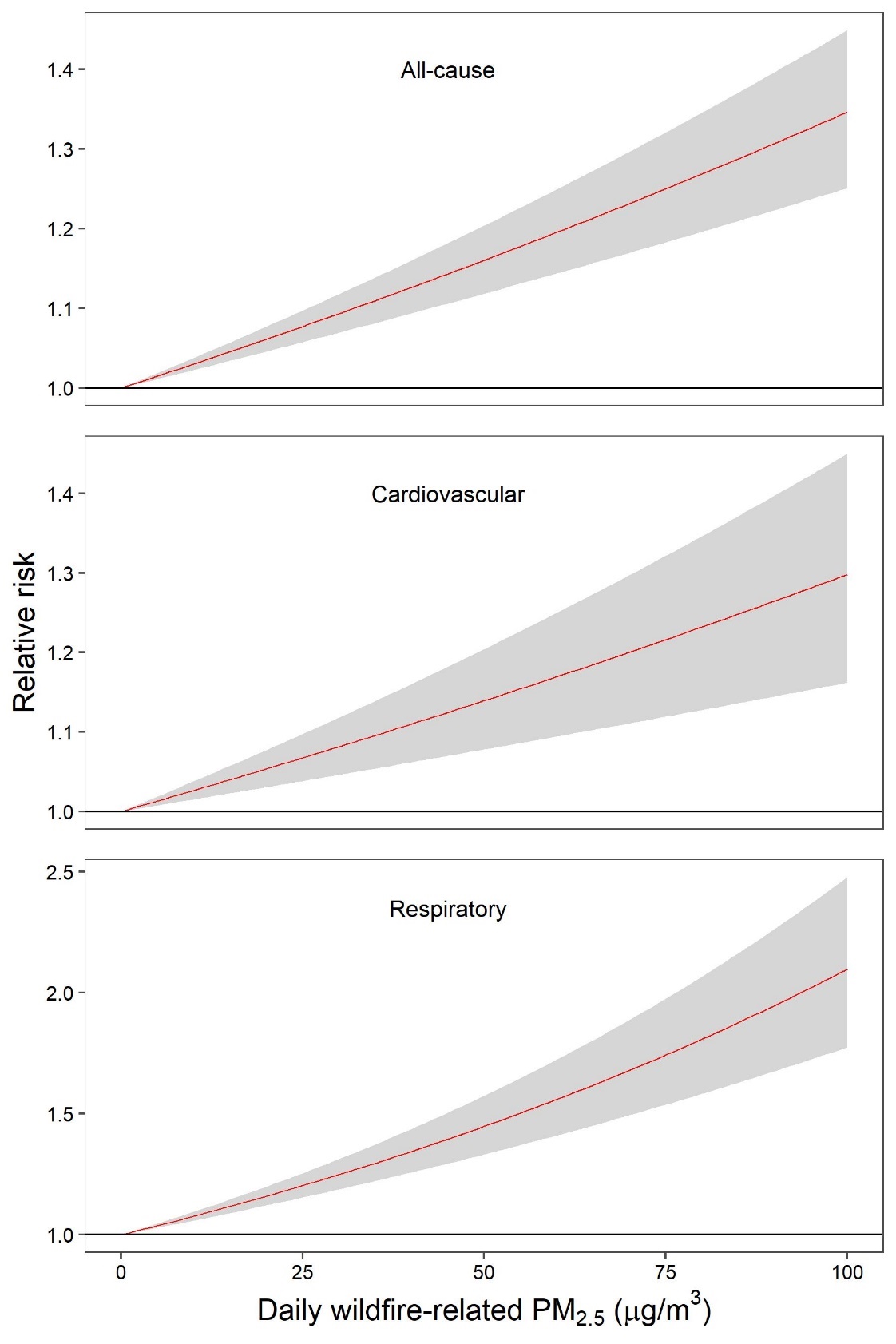


**Supplementary Figure 2**. The linearity of the relationship between daily wildfire-related PM_2.5_ and all-cause, cardiovascular, and respiratory mortalities across lag 0–7 days. (Note 1: The solid lines denote point-estimates and shaded areas denote the corresponding 95% conﬁdence intervals. Note 2: PM_2.5_, fine particulate matter with diameter ≤ 2.5 μm).

**Supplementary Figure 3.** The sex differences within each age subgroup. Pooled relative risks associated with a 10 µg/m^3^ increase in wildfire-related PM_2.5_ over lag 0‒14 days. (Note 1: The overall estimate within each age subgroup is the effect estimate based on the complete sample, not the pooled result of subgroup analyses by meta-analysis. Note 2: *p* value for the differences in cumulative relative risks (with 95% CI) across population subgroups were estimated by fixed effect meta-regression. Note 3: Black error bars correspond to 95% confidence intervals, center for the error bars correspond to points estimate of RRs. Note 4: Cause-, age- and sex-specific estimates were derived from the main model with data in 510 immediate regions. Note 5: PM_2.5_, fine particulate matter with diameter ≤ 2.5 μm).





**Supplementary Figure 4**. Lag patterns with different maximum lag day. The association between wildfire-related PM_2.5_ exposure (every 10 μg/m^3^ increase in wildfire-related PM_2.5_) and all-cause, cardiovascular, and respiratory mortalities across 0–14 lag days represented as relative risk (RR). Note 1: The solid lines denote point-estimates and shaded areas denote the corresponding 95% conﬁdence intervals. Note 2: Estimates were derived from models with data in 510 immediate regions. Note 3: PM_2.5_, fine particulate matter with diameter ≤ 2.5 μm).

**Single lag coefficients** $\boldsymbol{\beta}$**, and cumulated coefficients** $\hat{\boldsymbol{\beta}}$ **via model (1)**

**Pooled immediate-region-specific effects by meta-analysis as** $\boldsymbol{\beta'}$**,** $\hat{\boldsymbol{\beta'}}$

**Converted to with every 10 μg/m^3^ increase in wildfire-related PM_2.5_, i.e.,** $\boldsymbol{RR}$**,** $\hat{\boldsymbol{RR}}$

**Converted to immediate-region-specific relative risks (RR),** $\boldsymbol{RR}_{\boldsymbol{ti}}\boldsymbol{=exp (}\boldsymbol{\beta}_{\boldsymbol{i}}\boldsymbol{\times\Delta}\boldsymbol{x}_{\boldsymbol{ti}}\boldsymbol{)}$

**Calculated attributable health burden for each immediate region.** $\boldsymbol{AD}_{\boldsymbol{ti}}\mathbf{=}\boldsymbol{D}_{\boldsymbol{ti}}\boldsymbol{\times(}\boldsymbol{RR}_{\boldsymbol{ti}}\mathbf{-1)/}\boldsymbol{RR}_{\boldsymbol{ti}}$

**Results presented in Figure 2, Figure 3**

**Results presented in Table 2**

**Supplementary Figure 5.** The framework of statistical analysis. **(**Note: PM_2.5_, fine particulate matter with diameter ≤ 2.5 μm).

**Supplementary Table 1.** Results of sensitivity analyses changing maximum lag days and the df of lag days for wildfire-related PM_2.5_, and the df of lag days for daily mean temperature.

| Models | Relative Risks  (95% CI) | *p*-value | *p*-value for difference* |
| --- | --- | --- | --- |
| Primary | 1.031 (1.024, 1.039) | <0.001 | Ref |
| Lag 0-13 days | 1.031 (1.024, 1.038) | <0.001 | 0.999 |
| Lag 0-15 days | 1.032 (1.024, 1.040) | <0.001 | 0.867 |
| Lag 0-16 days | 1.033 (1.026, 1.041) | <0.001 | 0.671 |
| df of lag days = 3 | 1.029 (1.022, 1.037) | <0.001 | 0.742 |
| df of lag days = 5 | 1.033 (1.026, 1.041) | <0.001 | 0.697 |
| df for meteorological variables = 4 | 1.031 (1.023, 1.038) | <0.001 | 0.931 |
| df for meteorological variables = 5 | 1.030 (1.023, 1.038) | <0.001 | 0.874 |

Note: df=degree of freedom. PM_2.5_, fine particulate matter with diameter ≤ 2.5 μm. Relative risks represent the overall associations between every 10 μg/m^3^ increase in wildfire-related PM_2.5_ over lag days and all-cause mortality. **P*-value for differences were estimated by fixed effect meta-regression.
