# Supplementary material for: Short-term exposure to wildfire-related PM2.5 increases mortality risks and burdens in Brazil
Source: Nat Commun. 2022 Dec 10;13:7651. doi: 10.1038/s41467-022-35326-x (PMC9741581; doi:10.1038/s41467-022-35326-x)
Supplement: Supplementary file 2 — Reporting Summary [file 41467_2022_35326_MOESM2_ESM.pdf]

nature portfolio

Corresponding author(s): Yuming Guo; Shanshan Li

Last updated by author(s): Nov 18, 2022

Reporting Summary

Nature Portfolio wishes to improve the reproducibility of the work that we publish. This form provides structure for consistency and transparency in reporting. For further information on Nature Portfolio policies, see our Editorial Policies and the Editorial Policy Checklist.

Statistics

For all statistical analyses, confirm that the following items are present in the figure legend, table legend, main text, or Methods section.

n/a	Confirmed	
☐	☒	The exact sample size (*n*) for each experimental group/condition, given as a discrete number and unit of measurement
☒	☐	A statement on whether measurements were taken from distinct samples or whether the same sample was measured repeatedly
☐	☒	The statistical test(s) used AND whether they are one- or two-sided<br>*Only common tests should be described solely by name; describe more complex techniques in the Methods section.*
☐	☒	A description of all covariates tested
☐	☒	A description of any assumptions or corrections, such as tests of normality and adjustment for multiple comparisons
☐	☒	A full description of the statistical parameters including central tendency (e.g. means) or other basic estimates (e.g. regression coefficient) AND variation (e.g. standard deviation) or associated estimates of uncertainty (e.g. confidence intervals)
☐	☒	For null hypothesis testing, the test statistic (e.g. *F*, *t*, *r*) with confidence intervals, effect sizes, degrees of freedom and *P* value noted<br>*Give P values as exact values whenever suitable.*
☒	☐	For Bayesian analysis, information on the choice of priors and Markov chain Monte Carlo settings
☐	☒	For hierarchical and complex designs, identification of the appropriate level for tests and full reporting of outcomes
☒	☐	Estimates of effect sizes (e.g. Cohen's *d*, Pearson's *r*), indicating how they were calculated

Our web collection on statistics for biologists contains articles on many of the points above.

Software and code

Policy information about availability of computer code

Data collection	We collected hourly surface temperature and ambient dew point temperature from the European Centre for Medium-Range Weather Forecasts Reanalysis, v5 (ERA5) via Google Earth Engine platform. We then calculated daily mean relative humidity from the ERA5 daily mean temperature and daily mean dew point temperature, using the algorithm provided by the "humidity (version 0.1.5)" R package.
Data analysis	All data analyses were performed using R software (version 3.6.1). The "dlnm (version 2.4.7)" package was used to fit a distributed lag linear model, and the "mvmeta (version 1.0.3)" package to fit meta-analysis and meta- regression.

For manuscripts utilizing custom algorithms or software that are central to the research but not yet described in published literature, software must be made available to editors and reviewers. We strongly encourage code deposition in a community repository (e.g. GitHub). See the Nature Portfolio guidelines for submitting code & software for further information.

Data

Policy information about availability of data

All manuscripts must include a data availability statement. This statement should provide the following information, where applicable:

- Accession codes, unique identifiers, or web links for publicly available datasets
- A description of any restrictions on data availability
- For clinical datasets or third party data, please ensure that the statement adheres to our policy

The authors are not permitted to share the third party raw data used in the analyses. Population data can be downloaded from the website of the Brazilian Institute of Geography and Statistics (BIGS, https://www.ibge.gov.br/pt/inicio.html). Modern-Era Retrospective analysis for Research and Applications version 2 (MERRA-2)

data, biomass burning emissions inventory of Global Fire Emissions Database version 4.1 (GFED V4.1) data, and anthropogenic emissions inventory of EDGAR version 4.2 data that support the GEOS-Chem model development and wildfire-related PM2.5 simulation in this study are available from https://gmao.gsfc.nasa.gov/reanalysis/MERRA-2/, https://daac.ornl.gov/VEGETATION/guides/fire_emissions_v4_R1.html, and http://edgar.jrc. ec.europa.eu/, respectively. The hourly surface temperature and ambient dew point temperature from the European Centre for Medium-Range Weather Forecasts Reanalysis, v5 (ERA5) are available from https://www.ecmwf.int/en/forecasts/datasets/reanalysis-datasets/era5. The base map of Figure 1A-D is available in the Brazilian of Geography and Statistics under this following link: https://www.ibge.gov.br/en/geosciences/territorial-organization/territorial-meshes/18890-municipal-mesh.html?=&t=o-que-e. Source data for the figures are provided under this following link: https://github.com/pipty/2022_Brazil_firePM2.5_mortality.

Human research participants

Policy information about studies involving human research participants and Sex and Gender in Research.

Reporting on sex and gender	We collected death records from 2000 to 2016 from the Brazil Mortality Information System (Sistema de Informação sobre Mortalidade, SIM) (doi: 10.1590/0103-11042017s09). Each death record included information on individual's sex.
Population characteristics	see above
Recruitment	does not apply
Ethics oversight	Ethical approval was not required for this analysis of anonymous data.

Note that full information on the approval of the study protocol must also be provided in the manuscript.

Field-specific reporting

Please select the one below that is the best fit for your research. If you are not sure, read the appropriate sections before making your selection.

☐ Life sciences ☐ Behavioural & social sciences ☒ Ecological, evolutionary & environmental sciences

For a reference copy of the document with all sections, see nature.com/documents/nr-reporting-summary-flat.pdf

Ecological, evolutionary & environmental sciences study design

All studies must disclose on these points even when the disclosure is negative.

Study description	Data of death records from the National Unified Health System were collected from 2000 to 2016. Daily average wildfire-related PM2.5 for 510 immediate regions in Brazil were modeled during the same period. A two-stage time-series approach with quasi-Poisson regressions was used to assess risks and burdens of respiratory, cardiovascular, and all-cause mortality associated with short-term exposure to wildfire-related PM2.5.
Research sample	A total of 18,681,906 death records were collected from the National Unified Health System. Each death record included information on individual's municipality, age, sex, death date and primary cause of death coded according to the International Statistical Classification of Diseases and Related Health Problems, 10th Revision (ICD-10). Among them, 57.3 % were males and the ages ranged from 0 to 99 years, were then grouped into two age groups (i.e., 0–59 years, ≥ 60 years). This national dataset represents Brazil well.
Sampling strategy	No sample size calculation was performed. We used a nationwide dataset which is sufficient to represent population.
Data collection	Data were downloaded and organized by RX, PY, and TY. Grouped daily death counts into immediate region level.
Timing and spatial scale	Data were from 2000 to 2016. There was not a gap between collection periods. Regarding the spatial scale, data from 5574 municipalities were grouped according to immediate regions devisions in Brazil. In total, there are 510 immediate regions. Thus, this dataset has has a national coverage.
Data exclusions	For cause-specific analysis, we excluded death records without ICD code for cardiovascular and respiratory mortality, respectively.
Reproducibility	N/A. This study is an observational study.
Randomization	N/A. This study is an observational study.
Blinding	N/A. This study is an observational study.

Did the study involve field work? ☐ Yes ☒ No

Reporting for specific materials, systems and methods

We require information from authors about some types of materials, experimental systems and methods used in many studies. Here, indicate whether each material, system or method listed is relevant to your study. If you are not sure if a list item applies to your research, read the appropriate section before selecting a response.

Materials & experimental systems

n/a	Involved in the study
☒	☐ Antibodies
☒	☐ Eukaryotic cell lines
☒	☐ Palaeontology and archaeology
☒	☐ Animals and other organisms
☒	☐ Clinical data
☒	☐ Dual use research of concern

Methods

n/a	Involved in the study
☒	☐ ChIP-seq
☒	☐ Flow cytometry
☒	☐ MRI-based neuroimaging
